# The Impact of Metabolic Syndrome and Type 2 Diabetes *Mellitus* on Prostate Cancer

**DOI:** 10.3389/fcell.2022.843458

**Published:** 2022-03-25

**Authors:** André P. Sousa, Raquel Costa, Marco G. Alves, Raquel Soares, Pilar Baylina, Rúben Fernandes

**Affiliations:** ^1^ LaBMI-Laboratório de Biotecnologia Médica e Industrial, Porto, Portugal; ^2^ Department of Biomedicine, Unit of Biochemistry, Faculty of Medicine of Porto University, Porto, Portugal; ^3^ I3S-Instituto de Investigação e Inovação em Saúde, Porto, Portugal; ^4^ ICBAS-Instituto de Ciências Biomédicas Abel Salazar, Porto, Portugal; ^5^ ESS-Escola Superior de Saúde, Instituto Politécnico do Porto, Porto, Portugal

**Keywords:** metabolic syndrome, diabetes, prostate cancer, metabolism, signaling/signaling pathways, cancer biology

## Abstract

Prostate cancer (PCa) remains the second most common type of cancer in men worldwide in 2020. Despite its low death rate, the need for new therapies or prevention strategies is critical. The prostate carcinogenesis process is complex and multifactorial. PCa is caused by a variety of mutations and carcinogenic events that constitutes the disease’s multifactorial focus, capable of not only remodeling cellular activity, but also modeling metabolic pathways to allow adaptation to the nutritional requirements of the tumor, creating a propitious microenvironment. Some risk factors have been linked to the development of PCa, including Metabolic Syndrome (MetS) and Type 2 Diabetes *Mellitus* (T2DM). MetS is intrinsically related to PCa carcinogenic development, increasing its aggressiveness. On the other hand, T2DM has the opposite impact, although in other carcinomas its effect is similar to the MetS. Although these two metabolic disorders may share some developmental processes, such as obesity, insulin resistance, and dyslipidemia, their influence on PCa prognosis appears to have an inverse effect, which makes this a paradox. Understanding the phenomena behind this paradoxical behavior may lead to new concepts into the comprehension of the diseases, as well as to evaluate new therapeutical targets. Thus, this review aimed to evaluate the impact of metabolic disorders in PCa’s aggressiveness state and metabolism.

## Introduction

Currently, there is no universally accepted definition of cancer. However, it can be defined as uncontrolled cell development that results in a non-predetermined function and the ability to invade or metastasize to other body locations (Wang et al., 2018; Hausman, 2019). This metastatic behavior constitutes the key factor that reflects the difference between benign or malignant tumors since benign tumors remain at the primary tumor site, but malignant tumors infiltrate surrounding tissues and metastasis once they reach blood vessels (Wang et al., 2018).

Normally, cancer is not induced by external organisms such as viruses, bacteria, or parasites, though this can happen in some cases, such as cervical cancer (Hausman, 2019). The accumulation of unrepaired mutations is the primary cause of tumor formation, as it leads to not only genotype changes that allow rapid proliferation and avoidance of apoptosis, but also phenotype changes (Hausman, 2019). Besides that, several risk factors can increase the likelihood of cancer developing—tobacco, alcohol consumption, infections, a poor diet, a sedentary lifestyle, radiation, and, in particular, type 2 diabetes *mellitus* and metabolic syndrome are among them (Avgerinos et al., 2019; Hulvat, 2020).

According to WHO, cancer is the world’s second leading cause of mortality, with 9.6 million deaths and 18.1 million cases per year (Avgerinos et al., 2019; Hulvat, 2020). However, this is changing, since cancer has just been verified as the major cause of death in developed countries, with cancer set to overtake heart disease as the leading cause of death by 2030 (Mattiuzzi and Lippi, 2019). In men, lung, prostate, and colorectal cancers have the highest risk of malignancy compared to other carcinomas (Mattiuzzi and Lippi, 2019). According to GLOBOCAN, there would be 27.5 million new cancer cases in 2040, representing a 61.7% rise. The number of cancer survivors has increased as a result of new cancer detection tools and treatments (Hulvat, 2020).

Prostate cancer (PCa) is a heterogeneous disease, and it is the third most frequent malignancy in men worldwide (behind lung and colorectal cancers) with 1.4 million diagnoses and roughly 360 thousand deaths each year according to data from 2020 (Bott and Ng, 2015). The number of diagnoses appears to be influenced by the Human Development Score (the higher the index, the higher the frequency of PCa), as well as by rising age (average age of diagnosis is 66 years) (Rawla, 2016; Bray et al., 2018). Furthermore, specific regions around the world tend to have a positive or negative relationship with PCa, with higher frequency in Europe, the Caribbean, Australia, North America, and Southern Africa (Bott and Ng, 2015). Differences in social, environmental, and genetic factors have been proposed as causes for this disparity. Even though 2.3 million new cases are projected till 2040, there will be a slight fluctuation in mortality, bringing the total number of fatalities to around 380 thousand (Rawla, 2016).

PCa is often misdiagnosed as prostatic hypertrophy in its early stages, when the symptoms of both are frequent and difficult urination, as well as nocturia which can develop to worst-case scenarios such as back discomfort with urine retention and, in PCa cases, metastatic progression to bone (Rawla, 2016). Diet and physical activity, as well as the tumor’s location in the body, appear to have a direct impact on its progression (Rawla, 2016).

### Prostate Cancer Etiology

The prostate is a glandular-structured, embryologically derived of the urogenital sinus in the male reproductive system. It is responsible for including an alkaline fluid rich in nutrients that enriches and preserves the ejaculated semen, promotes healthy ejaculation, and stimulates fertility. Because of its great susceptibility to carcinogenesis due to inappropriate expression of some signaling pathways, it has a considerably higher incidence than other urogenital structures (Bott and Ng, 2015).

**FIGURE 1:**
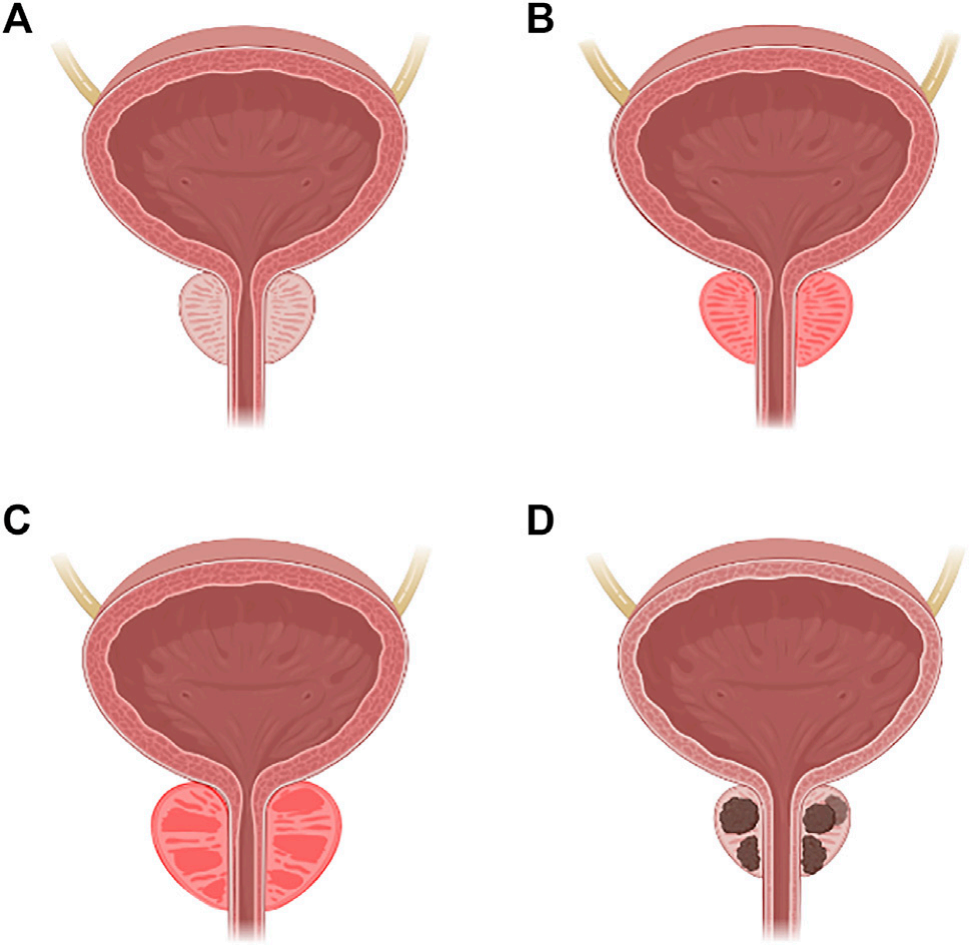
Carcinogenesis of the prostate. **(A)** Normal prostate. **(B)** Prostatitis. **(C)** Benign prostatic hyperplasia. **(D)** Prostatic carcinoma.

Normal prostate tissue can develop into PCa through a mechanism that will be further discussed thoroughly; however, several pathological states have been described before the carcinoma transition that is depicted in Figure 1, including prostatitis, benign prostatic hyperplasia, and prostatic carcinoma (Sharp et al., 2010).

Prostatitis is an inflammatory condition of prostate tissue that has different stages. Acute bacterial prostatitis (category 1), is characterized by an acute bacterial infection caused by Gram-negative (*Escherichia coli, Klebsiella* spp.*, Proteus* spp.*, Pseudomonas* spp.) or Gram-positive (*Enterococcus* spp.) bacteria, which can progress to a prostatic abscess or sepsis if untreated (Sharp et al., 2010). Chronic bacterial prostatitis (category 2) is defined as an infection that lasts longer than 3 months and is considered to be caused by bacteria’s ability to form biofilms that make them less sensitive to therapy, being the same microorganism detected in multiple diagnoses (Sharp et al., 2010). Chronic prostatitis or chronic pelvic pain syndrome (category 3) is a continuation of the previous category, with the exception that depending on the presence of leukocytes in the area, it may or may not be inflammatory (Sharp et al., 2010). Finally, the fourth and last category is asymptomatic prostatitis, where the diagnosis is made when leukocytes are identified in the sperm or through urologic assessment (Sharp et al., 2010).

Benign prostatic hyperplasia is a nonmalignant growth of the prostate caused by an increase in the number of epithelial or stromal cells, which results in nodules that can obstruct the urethra and cause severe pain. Nocturia, weak and infrequent urination, urgency, and discomfort are all symptoms that are similar to prostatitis. The prevalence of this disease is also age-related (Bachmann, 2015).

Prostatic carcinoma is expected linked to embryological organogenesis, as previously stated, along with its relation to androgenic hormone signaling or other altered growth and proliferation pathways (Rawla, 2016). PCa can be caused by sarcoma/mesenchymal or lymphoma, in alternative to the most prevalent malignancy in the epithelial layer. There is a wide variety of research going on to establish which cell type is responsible for oncogenesis and how their origin can lead to new therapeutic options (Rawla, 2016).

The most frequent PCa described is prostatic intraepithelial neoplasia, which is the rapid growth of a pre-existing benign epithelium, and it can be divided into a low or high grade (only detected by needle biopsy, once another diagnosis is not available for this specific type) (Rawla, 2016).

Recent research has focused on identifying the genes and mutations that cause PCa, and knowing the risk factors that can influence this process, as well as the etiology, may contribute to finding new therapeutical approaches (Bray et al., 2018). Although the main etiology remains a gap in the scientifical community, there are some risk factors positively associated with PCa pathology, including age, ethnicity, treatment, ejaculatory frequency, sexually transmitted diseases, genetic factors, diet, smoking, sexual hormones, hyperglycemic state, chronic inflammation, obesity, and hyperinsulinemia (Adjakly et al., 2015).

#### Age

Due to increased life expectancy, which provides a set of mutations/susceptibilities, the elderly population is the most impacted by PCa (Rawla, 2016; Bray et al., 2018).

#### Ethnicity

PCa prevalence appears to be influenced by racial groups such as African, British, and Caribbean men. It seems to be not only by biological causes but also by socioeconomic demography that allows a more sedentary lifestyle (Adjakly et al., 2015; Rawla, 2016).

#### Treatment—Medications, Vasectomy, and Radiological Exposure

Vasectomy, the most common male contraception procedure, has inconsistent results when it comes to PCa progression, although it appears to have been a potential risk factor. Furthermore, some carcinogens in the environment, drugs, and radiation exposure appear to have a harmful impact on PCa (Adjakly et al., 2015).

#### Ejaculatory Frequency

Scientific evidence point out that lower chances of development of PCa are related to a higher ejaculatory recurrence (Adjakly et al., 2015).

#### Sexually Transmitted Diseases

When it comes to STDs, there is agreement since scientific evidence reveals that risky sexual behavior and STDs increase the risk of developing PCa, particularly gonorrhea and syphilis. Thus, a more active sexual life may promote PCa due to the increased probability to become infected by STD (Leitzmann and Rohrmann, 2012).

#### Genetic Factors

In families with a history of PCa, common lifestyle choices and shared paternal genes may have a role. Some gene mutations, such as in the enzyme ribonuclease L (innate immune defense mechanisms against a viral infection), ELAC2 Zinc phosphodiesterase (endonuclease possibly involved in mitochondrial tRNA maturation), macrophage scavenger receptor 1 (endocytosis of modified low-density lipoproteins), BRAC1/BRAC2 and in chromosome X (since it contains the gene of androgen receptors), have been reported to transfer down through generations and enhance the probability of PCa development (Rawla, 2016).

#### Diet

Saturated animal fat, red meat, and soy appear to be linked to the PCa process, increasing the probability of its development. Furthermore, some vitamins and minerals such as calcium, vitamin D and E, folate, and vitamin B12, as well as certain vegetables, milk and green tea have a questionable relationship with PCa, but a protective effect against it has been reported (Adjakly et al., 2015).

Alcoholic beverages and coffee, on the other hand, have a more direct effect on PCa. Coffee plays a protective role against PCa, whereas alcoholic beverages tend to be a significant risk factor for the development of a variety of cancers, including PCa (Rawla, 2016).

#### Smoking

This activity, whether passive or active smoking, is a potential risk factor for PCa since it affects hormonal (increased circulating sexual hormones that lead to PCa) and genetic bases (the polycyclic aromatic hydrocarbons metabolism affect progression and initiation of general carcinomas) (Adjakly et al., 2015; Bott and Ng, 2015).

#### Sexual Hormones

Huggins and Hodges proposed the androgen theory, which establishes the link between PCa and the role of androgens in its progression, with androgen withdrawal leading to PCa diminishment *in vivo* or *in vitro*. Once single nucleotide polymorphisms in the genes of hydroxysteroid, dehydrogenase-1, hydroxy-delta-5-steroid dehydrogenase, 5α-reductase-1 and -2, testosterone, dihydrotestosterone, estrogen receptors, CYP17, CYP3A4, and CYP19A1 were linked to the androgen signaling pathway, it was thought to be one of the most important pathways regulating PCa (Rawla, 2016).

Although scientific evidence suggests that androgen deprivation reduces PCa, several inconsistencies have been discovered, including estradiol being identified as a carcinogenic component (Bott and Ng, 2015).

#### Hyperglycemic State

Although hyperglycemia is linked to potentiate most malignancies, controversial results were found in PCa. Hyperglycemia has been associated to have a protective effect in PCa even though glucose has been linked to tumor development as a quick source of energy. In this type of cancer, other mechanisms such as oxidative stress, apoptosis, DNA damage, and chronic inflammation are all caused by this glucose environment and may overlap the capacity of the energy source (Rawla, 2016).

#### Chronic Inflammation

When substantial numbers of leukocytes were discovered in carcinoma biopsies, a link between PCa and inflammation was discovered. This results in prostatic intraepithelial neoplasia, which has been regarded as the most common type of PCa (Rawla, 2016).

#### Obesity, Insulin, and Physical Activity

Obesity has a positive relationship with PCa since it promotes more aggressive and severe carcinomas, and higher body mass index (BMI) values contribute to that mechanism. This could be due to changes in the amounts of metabolic and sexual steroid hormones, which enhance PCa. In this approach, physical activity may compensate for the effects of obesity, reversing some of the detrimental mechanisms that lead to PCa development (Leitzmann and Rohrmann, 2012; Bott and Ng, 2015; Rawla, 2016).

When these risk factors are combined with epigenetics, the circumstances for PCa development are generated. As a result, it’s vital to understand the disease’s molecular pathways, as well as how they affect metabolism in general. The modified pathways not only cause the activation of some malignant mechanisms, such as increased proliferation but also cause the cell to adapt to the cancer microenvironment, allowing for surveillance and adaptability (Hughes et al., 2005).

## PCa Molecular Pathways

### Genetic and Signaling Dysregulations

PCa can be classified into hereditary and sporadic cancers based on epidemiology, albeit molecular interpretation makes this distinction untenable (Hughes et al., 2005). Some research supports some inherent alleles in PCa and how alterations in particular genes make the prostate more sensitive to developing carcinomas (Hughes et al., 2005).

#### Hereditary PCa

Some susceptibility genes have been identified in this type of cancer; however, the number of instances of PCa linked to these specific susceptibilities is quite small, and the results are still ambiguous (Hughes et al., 2005). Furthermore, several mutations have been described in sporadic PCa, and more research is needed to fully understand this information. Additionally, the genes involved in hereditary PCa appear to have a minor impact on its progression, implying that other gene changes are required to progress malignancy (Hughes et al., 2005).

##### Zinc Phosphodiesterase ELAC Protein 2

ELAC2, which participates in the maturation of mitochondrial tRNA, was the first gene discovered as a probable hereditary gene. Nevertheless, it plays a minimal function in the development of PCa (Hughes et al., 2005; Rawla, 2016).

##### Ribonuclease L (RNASEL or HPC1)

This gene, also known as Hereditary Prostate Cancer 1, which encodes a ribonuclease L that causes the death of viruses and cells by degrading their RNA, is the most important and critical gene involved with hereditary PCa (Hughes et al., 2005).

##### Predisposing for Prostate Cancer

PCAP is a region in chromosome 1 and was the second location to be associated with the PCa development (Mazaris and Tsiotras, 2013).

##### 3β-Hydroxysteroid Dehydrogenase

HSD3B is a region from chromosome 1 that may have a higher influence in PCa development, with no established function, subsequently more research is required (Mazaris and Tsiotras, 2013).

##### Carcinoma Prostate Brain

CAPB is a chromosome 1 region that establishes a positive association between PCa and human brain cancer development. According to research, it plays a significant part in both types of cancer (Mazaris and Tsiotras, 2013).

##### Hereditary Prostate Cancer 20

HPC20 is a chromosome 20 location with a high predisposition to mutation and develop PCa, and its function is still being under investigation (Mazaris and Tsiotras, 2013).

##### Hereditary Prostate Cancer X-Linked

HPCX is another region, but now on chromosome X, that may be associated with hereditary prostate cancer (Mazaris and Tsiotras, 2013).

##### Macrophage Receptor Scavenger 1

This gene encodes macrophage scavenger receptor, which regulates the uptake of compounds from, for example, bacteria. Its role in PCa is questionable, although reports reveal that some families have mutations in this gene, which can contribute to persistent inflammation, which can lead to PCa (Hughes et al., 2005).

##### Nijmegen Breakage Syndrome 1 Gene

Nijmegen breakage syndrome is a rare genetic disorder that produces protein nibrin, a protein involved in the repair and processing of DNA as well as in cell cycle checkpoints. Mutations in this gene cause the host to become immunodeficient, radiosensitive, acquires chromosomal instability and increases the risk of lymphatic cancer. This gene appears to have a slight effect on sporadic cancer as well as hereditary cancer, thus mutations lead to a low increase in the probability of developing PCa (Hughes et al., 2005).

##### Checkpoint Kinase 2

CHEK2 gene participates in DNA damage signaling pathway, regulating p53 gene. Sporadic and hereditary cancer appears with mutations in this gene and is associated with a low increase of PCa development. Its mechanism of action remains unclear (Hughes et al., 2005).

Table 1 summarizes the effect of each gene in PCa development, as well as its influence to modulate the progression and aggressiveness of PCa.

**TABLE 1:** Common hereditary mutations in PCa development. The normal function of each gene is represented, along with its impact in PCa development.

Gene	Function	Influence on PCa carcinogenesis
ELAC2	Maturation of mitochondrial tRNA	Minimal
RNASEL or HPC1	Degradation of external RNA sources	Critical
PCAP	Loci with PCa predisposition	Undefined
HSD3B	Loci with PCa predisposition	Critical
CAPB	Brian and prostate carcinogenesis	Critical
HPC20	Loci with PCa predisposition	Undefined
HPCX	Loci with PCa predisposition	Undefined
MRS1	Degradation of external biomolecules	Minimal
NSB1	Regulation of DNA repairing and cell cycle checkpoints	Minimal
CHEK2	Regulation of DNA repairing	Minimal

#### Sporadic PCa

The overwhelming majority of PCa cases are sporadic and unrelated to hereditary factors. Some gene mutations have been identified, as well as dysregulations in signaling pathways involved in PCa development (Hughes et al., 2005). There are six different types of carcinogenic phenomena that, in general, promote the progression of PCa.

#### Telomere Shortening

Replication tends to lead to a natural shortening of telomeres as a result of aging in biochemically stable cells. However, these telomeres did not appear to be shortening in cancers in general, and notably in PCa. According to scientific research, this occurrence is attributed to the fact that the enzyme telomerase, an enzyme that prevents telomere degradation, is with high activity, thus increasing the longevity of tumor cells (Hughes et al., 2005).

#### Polymorphisms

Polymorphisms are low-risk genetic variants that impact more than 1% of the population and are linked to disease development due to their high prevalence (Hughes et al., 2005; Mazaris and Tsiotras, 2013; Coleman et al., 2018). Some authors such Hughes et al. (2015), Mazaris et al. (2013) and Coleman et al. (2018) identified several polymorphisms that are correlated to PCa.

##### Androgen Receptor

Prostate and PCa development and growth rely on androgens (Bott and Ng, 2015). This gene includes trinucleotide repeats of polyglutamine (CAG) that, when shortened, increase PCa risk, aggressiveness, grade, metastatic potential and mortality, although literature shows controversial results (Hughes et al., 2005).

The hypothalamic-pituitary-gonadal axis is the primary regulator of androgen production. Changes in this axis that result in a decrease in androgen serum levels, which are precursors to PCa, have made this axis a therapeutic target (Bott and Ng, 2015).

Activated by testosterone and dihydrotestosterone (DHT) which leads to differentiation, proliferation and apoptosis of epithelial prostate cells (Bott and Ng, 2015), AR is particularly crucial in PCa, to the point where blocking them is one of the most popular therapeutic approaches in cases where surgical removal is unfeasible or when it has metastatic. However, additional mutations on that gene may proceed to a state where PCa becomes AR independent to growth, resulting in the ineffectiveness of that therapeutical method and the rise of castration-resistant PCa (CRPC), a considerably more aggressive cancer type (Hughes et al., 2005; Bott and Ng, 2015).

In CRPC, other molecular pathways are activated to fulfill the needs of PCa cells and allow them to proliferate and grow, including growth factor-β (TGF-β), epidermal growth factor (EGF), fibroblast growth factor (FGF) and insulin-like growth factor (IGF), all establishing a connection nominated as “cross-talk” (Bott and Ng, 2015).

This hyper sensibilization of some pathways activates several molecular mechanisms in the cell to promote cell survival, highlighting the mutation from protein phosphatase and tensin homolog gene (PTEN) and consequent activation of phosphatidylinositol 3-kinase and consequently PI3K-AKT-mTOR pathway, which inhibits autophagy and improves cell cycle progression (Bott and Ng, 2015).

##### Toll Like Receptor 4

TLR4 is a gene involved in the innate immune response to Gram-negative bacteria, and mutations in this gene have been linked to a low increase in PCa risk, corroborating previous findings that inflammation is a key factor in PCa development (Hughes et al., 2005).

##### Cytochrome P-450c17a

CYP17 is a crucial enzyme in testosterone production, and literature shows that the most prevalent mutations in this gene result in increased expression, which leads to more testosterone production, and as a result more androgens, which is a major risk factor for PCa progression (Hughes et al., 2005).

##### Steroid 5α-Reductase 2

This gene codifies the enzyme responsible for the conversion from testosterone to a more potent form, which is dihydrotestosterone. When mutations are observed in this gene, a poor prognosis is commonly associated (Hughes et al., 2005).

#### Tumor Suppressor Genes

According to the literature, this kind of gene plays an important role in any cancer development. They are responsible to suppress tumorigenic mechanisms by inhibiting them, and their loss occurs normally by mutation or deletion that leads to a function loss on both alleles (Hughes et al., 2005; Coleman et al., 2018).

##### Glutathione S-Transferase

The GSTP1 protein protects cells from electrophilic carcinogens and oxidants, which are common DNA-damaging agents. According to recent findings, hypermethylation of the promoter region is one of the most important mutational events that increases the risk of PCa development. This gene has also been identified as one of the most frequently mutated genes, making it a viable diagnostic biomarker (Hughes et al., 2005).

##### Phosphatase and Tensin Homolog

This gene is one of the most important since it is involved in many signaling pathways, including the regulation of the PI3K-AKT-mTOR pathway and the expression of the p27 gene, which results in the cell blocking on G1 phase of the cell cycle, inhibiting proliferation, and improving AR gene expression (Bott and Ng, 2015). PTEN levels in PCa cells are significantly lower than in normal epithelial prostate cells due to mutations found in 20% of cases, according to the findings. This mutation is one of the mechanisms of the earlier CRPC transition, in which cells become androgen insensitive and adopt the PI3K-AKT-mTOR pathway as a proliferative pathway (Hughes et al., 2005; Bott and Ng, 2015).

##### Cyclin Dependent Kinase Inhibitor 1B or tp27

Lower levels of the functional protein p27 owing to mutations in this gene are associated with a poor prognosis; mutations are discovered in roughly 23% of PCa cases and more than double in metastatic cases (47%). A PTEN mutation may lead to a reduction of its expression since the activity from this gene is inversely controlled by the PI3K-AKT-mTOR pathway (Hughes et al., 2005).

##### Cyclin-Dependent Kinase Inhibitor 2A or tp16

The metastatic event in PCa appears to be linked to mutations in this gene (Mazaris and Tsiotras, 2013).

##### MX11

The MX11 gene is a c-MYC proto-oncogene inhibitor that is abundant in PCa. Mutations on it may increase the likelihood of PCa development (Mazaris and Tsiotras, 2013).

##### Homeobox Protein NKX3.1

Along with its early occurrence, mutations in this gene appear to be another possible biomarker for PCa. It has a role in normal prostate development by blocking the gene that produces prostate-specific antigen (Hughes et al., 2005).

##### Krueppel-Like Factor 6

Losses and deletions in this gene, which is linked to carcinogenesis processing, have been associated with the progression of PCa to a worse prognosis, according to reports (Hughes et al., 2005).

##### Retinoblastoma

Rb gene mutations are involved in a substantial amount of malignancies by their role in the G1 phase of the cell cycle (Hughes et al., 2005).

##### Tumor Protein 53

This gene is responsible to drives cells into the apoptotic process when DNA has been permanently damaged. This way, loss of its function affects PCa by promoting uncontrolled growth along with potentiating bone metastasis and androgen independence (Hughes et al., 2005).

##### Atrial Fibrillation Familial 1

Another gene implicated in cell cycle regulation is ATFB1. Mutations in it were reported in several PCa cases (Mazaris and Tsiotras, 2013).

##### Annexin

According to the literature, decreased expression of this gene in PCa causes disruptions in calcium homeostasis and cell motility, which is annexin’s role (Mazaris and Tsiotras, 2013).

##### Adenomatous Polyposis Coli

The Wnt/β-catenin pathway, which inhibits cell proliferation, is regulated by the APC gene. According to scientific evidence, increased methylation of these genes is critical for the onset of carcinogenesis and is directly proportional to the aggressiveness and development of PCa (Coleman et al., 2018).

#### Oncogenes

Oncogenes are mutated genes that play a role in tumorigenesis. In normal conditions, all cells present these genes in a nonmalignant state known as proto-oncogenes (Hughes et al., 2005). Some of these genes are directly related to PCa tumorigenesis.

##### c-MYC

This gene is linked to Gleason status, a grading system for tumors in which the higher the c-MYC expression, the worse the PCa prognosis (Hughes et al., 2005).

##### B-Cell Lymphoma 2

The BCL-2 gene family is responsible for preventing apoptosis (programmed cell death) by performing an anti-apoptotic role in mitochondria. In advanced stages of PCa, when the disease has progressed to CRPC, BCL-2 seems to play a significant role to lead to a worst prognosis (Hughes et al., 2005).

##### Tyrosine Kinase Receptor

The Src signaling pathway, responsible for numerous events that allow carcinogenesis to progress, is activated by oncogene c-Kit (Hughes et al., 2005).

##### Signal Transducer and Activator of Transcription 5

This oncogene is responsible for the activation of transcription and consequent protein translation to protein, which is an essential step to PCa survival. Poor prognosis was reported when STAT5 is upregulated (Hughes et al., 2005).

#### Growth Factors

These molecules are fundamental to maintain body homeostasis as well as to allow the development of any type of organ, including the normal functional prostate. Nevertheless, dysregulations on the expression of this factor may lead to irreversible phenomena that trigger tumorigenesis (Mazaris and Tsiotras, 2013).

##### Interleukin 6

This gene encodes a cytokine responsible for STAT activation, modulates apoptosis and regulates mitosis through activation of mitogen activated protein kinase (MAPK) signaling. High expression of IL-6 was found in PCa, and literature shows that this cytokine provides a microenvironment propitious to tumorigenesis, by inactivating other genes, for example, the Rb gene (Hughes et al., 2005).

##### Epidermal, Vascular Endothelial and Insulin Growth Factors

These growth factors increase angiogenesis, invasion, and metastasis in PCa by modulating the MAPK pathway (Hughes et al., 2005).

##### Epidermal Growth Factor Receptor (C-Erb-2 or HER2/Neu)

Literature shows that amplification of this growth factor along with neu glycoprotein enhances aggressiveness and metastasis probability, leading to higher grades of PCa and poor prognosis (Hughes et al., 2005; Mazaris and Tsiotras, 2013).

##### Factor/Nerve Growth Factor (Fas/Fas Ligand)

Apoptosis is avoided when this gene is dysregulated, which is a favorable environment for carcinogenesis (Hughes et al., 2005).

##### Hepatocyte Receptor Growth Factor (C-met)

The c-met gene is involved in embryogenesis, tissue organization, and tumor growth. Its expression has been linked to the worst prognosis in the literature, with higher levels of expression in androgen insensitivity and CRPC (Hughes et al., 2005).

##### Prostate Stem Cell Antigen

This gene seems to be related to the process of androgen independence of PCa, leading to more aggressive behavior. *In vitro* studies suggests this gene is a promising therapeutical target to this pathology (Mazaris and Tsiotras, 2013).

##### Erythroblast Transformation-Specific Related Gene and Transcription Variant 1

Both erythroblast transformation-specific genes are overexpressed in PCa, not only in the primary formation of the tumor but also in metastatic cells. Fusion to the TMPRSS2 gene, a prostate-specific cell-surface serine protease gene, appears to activate them, resulting in an androgen-responsive fusion oncoprotein (Mazaris and Tsiotras, 2013).

##### Hepsin

This gene encodes a membrane protein that is enrolled in cell growth, being related to PCa development (Mazaris and Tsiotras, 2013).

##### Serine/Threonine Kinase PIM1

It is a gene that is upregulated in PCa (Mazaris and Tsiotras, 2013).

##### A-Methyl Coenzyme a Racemase

As previously stated, red meat and saturated animal fat consumption are major risk factors for PCa onset, and the AMACR gene is responsible for fatty acid oxidation. Its mutations were found in the majority of clinical cases, suggesting that this gene could be employed as a diagnostic method for PCa (Mazaris and Tsiotras, 2013).

#### Invasion/Metastasis Suppressing Genes

Several genes have been found in the invasion and metastasis processes, where cells must be able to penetrate the surrounding stroma and vasculature, spread to new sites, and survive in new surroundings (Hughes et al., 2005). Activation/inhibition of some genes leads to worse grades of PCa, where treatments are less effective.

##### E-Cadherins

This membrane glycoprotein family gene is involved in cell-cell adhesion and recognition, and it is frequently altered in PCa to speed up the disease’s progression. Nonetheless, the findings are controversial, as other studies have found normal E-cadherin levels in PCa (Hughes et al., 2005).

##### Integrins

Integrins are required by prostate epithelial cells to maintain the layer’s structure; however, reports have shown that in PCa, this gene appears to be altered, and aberrant quantities of integrins have been discovered (Hughes et al., 2005).

##### Cell Adhesion Molecule

Mutations in this gene seem to be an early event in PCa, once normal epithelial cells interrupt its expression in cancer transition. The repositioning of this gene’s normal function is capable of reversing cancer growth and restoring normal function to the cell layer (Hughes et al., 2005).

##### CD82 or KAI1 Gene

This gene, along with others, appears to be one of the metastatic potential triggers. It is responsible for inhibiting metastatic progression under normal conditions, but mutations in PCa and other cancer types cause this gene to be overridden, allowing metastasis to begin (Hughes et al., 2005).

##### CD44

CD44 correlates to CD82 function, being linked to metastasis inhibition in normal settings (Hughes et al., 2005).

##### AMP-Activated Protein Kinase

This gene is regarded as the “guardian of cell metabolism” since it participates in a metabolic signaling pathway in which AMP and ADP, the low-energy and dephosphorylated forms of ATP, activate catabolic metabolism in order to restore ATP concentration. As a source of energy for processes, ATP is required by all cellular functions (Mamouni et al., 2021).

Glycolysis, lipid and mitochondrial homeostasis, and, most importantly, phosphorylating mTOR1 from the PI3K-AKT-mTOR pathway are all AMPK-dependent signaling pathways. In the autophagy process, which recycles macromolecules and organelles, AMPK and mTORC1 are regarded as antagonists. AMPK is active under starvation and causes autophagy to obtain nutrients; conversely, in normal circumstances, mTOR inhibits the AMPK signaling cascade and prevents autophagy (Mamouni et al., 2021). Table 2 summarizes the majority of genes involved in PCa development.

**TABLE 2:** Common somatic mutations in PCa development. The normal function of each gene is represented, along with its impact in PCa development.

Mutagenic phenomena	Gene	Function	Influence on PCa carcinogenesis
Telomerase activation	Telomerase	Telomere preservation; cell aging prevention	Critical
Polymorphisms	AR	Normal prostate and PCa growth signaling	Critical
	TLR4	Innate immune response	Minimal
	CYP17	Testosterone synthesis	Critical
	SRD5A2	Testosterone to dihydrotestosterone conversion	Critical
Tumor suppressor	GSTP1	Antioxidant defense	Critical
	PTEN	Inhibition of PI3K-AKT-mTOR pathway	Critical
	CDKN1B	Upregulated by PI3K-AKT-mTOR pathway	Critical
	CDKN2A	Metastatic event	Minimal
	MX11	Carcinogenesis inhibition	Minimal
	NKX3.1	PSA production	Critical
	KFL6	Carcinogenesis inhibition	Minimal
	Rb	Regulation of G1 phase of cell cycle	Minimal
	tp53	Regulation of DNA repairing	Critical
	ATFB1	Regulation of cell cycle	Critical
	Annexin	Cell motility	Critical
	APC	Cell proliferation	Critical
Oncogene	c-MYC	Tumor grade	Minimal
	BCL-2	Apoptosis inhibition	Critical
	c-KIT	Carcinogenesis	Minimal
	STAT5	Activator of gene transcription	Critical
Growth factors	IL-6	STAT activator; modulates mitosis and apoptosis	Critical
	Epidermal, vascular, insulin growth factors	Angiogenesis, invasion and metastasis	Critical
	HER2/neu	Aggressiveness and metastasis	Critical
	Fas/Fas ligand	Apoptosis regulation	Critical
	c-met	Cell growth and proliferation	Critical
	PSCA	AR pathway	Minimal
	ERG and ETV1	AR pathway	Critical
	Hepsin	Cell growth	Minimal
	Serine/Threonine Kinase	MAPK pathway	Critical
	AMACR	β-oxidation	Minimal
Invasion and metastasis	E-cadherins	Cell-cell recognition and adhesion	Critical
	Integrins	Layer structure	Critical
	C-CAM	Layer structure and integrity	Critical
	CD82	Metastasis inhibitor	Critical
	CD44	Metastasis inhibitor	Minimal
	AMPK	Major metabolism regulator	Critical

Although all these genes appear to play a role in the development of PCa, several interesting genes, such as GSTP1, NKX3.1, PTEN, and p27, have shown results that suggest they could be exploited as a diagnostic target. Other genes implicated in androgen synthesis, including AR, SRD5A2, and CYP17, are considered potential theragnostic molecules, suggesting they have the potential to act as disease biomarkers and therapeutic targets (Hughes et al., 2005).

According to the literature, the modifications that occur during PCa metabolism affect the entire central metabolism. Thus, it’s critical to comprehend the carcinogenic modeling process in metabolism and how it enables rapid tumor growth and proliferation.

### Metabolic Implications

The basal cellular metabolism uses glucose as an energy source, which occurs in two different ways: in anaerobiosis, a process that does not require oxygen, glucose is converted to pyruvate (glycolysis), which is then fermented directly, providing quick energy; in aerobiosis, oxygen is used as one of the mediators of large-scale energy production (Mamouni et al., 2021). However, Otto Warburg discovered that cells prefer to undertake anaerobic glycolysis even in the presence of oxygen, due to adaptation mechanisms that allow glucose to be used for other purposes such as amino acids and nucleotides production, which allow cell growth (Grasmann et al., 2019; Mamouni et al., 2021). However, it appears that the prostate has a unique metabolism that is not found in other tissues.

Normal prostate tissue is controlled by AR signaling, allowing it to produce prostatic fluid rich in zinc, lipids and citrate [an intermediate in the tricarboxylic acid (TCA) cycle that transports acetyl-CoA carbons to the cytoplasm for fatty acid production, energy storage, and membrane formation] (Mamouni et al., 2021). The cell relies on two mechanisms to generate large amounts of citrate: the first is that high levels of zinc in this tissue block aconitase, the enzyme responsible for converting citrate to isocitrate in the TCA cycle; the second is to rely on glycolysis to generate citrate without depending on TCA (Gillis et al., 2021). Nonetheless, AR signaling affects metabolism differently throughout carcinogenesis, and prostate cells gradually increase mitochondrial activity, using citrate as a fuel. As a result, the TCA cycle and oxidative phosphorylation increase, lowering glycolysis and citrate metabolism while increasing *de novo* lipogenesis (Mamouni et al., 2021).

#### Glycolysis

AR signaling appears to be important in glycolysis regulation, affecting enzymes including Glucose Transporter 1 (GLUT1) and Hexokinase 1 and 2 (HK1/HK2). In advanced stages of PCa, decreased glycolysis (Mamouni et al., 2021) and increased GLUT1 expression has been documented compared to normal cells (Kelly et al., 2016; Giunchi et al., 2019). This mechanism is generated not just by AR signaling, but also by IGF1 activation, which stimulates lower glucose absorption *via* the PI3K/AKT/mTOR pathway. Mutations in tp53 have been also reported to affect glucose metabolism by inactivating HK2 (a glycolysis enzyme that phosphorylates glucose to glucose-6-phosphate) and increasing GLUT1 expression as a compensatory mechanism (Kelly et al., 2016; Giunchi et al., 2019).

#### Pentose Phosphate Pathway

Another metabolic pathway that recruits glucose as a fuel is the pentose phosphate pathway, which produces pentoses and eventually ribose 5-phosphate, which is employed in the production of nucleotides. This mechanism generates a potent electron donner—NADPH—a molecule with high metabolic relevance since it participates in fatty acid synthesis and antioxidant defense by reducing glutathione (Gillis et al., 2021). As a result, PPP is a key pathway for maintaining cellular lipid and antioxidant homeostasis (Gillis et al., 2021).

The AR axis appears to upregulate the first PPP enzyme in PCa. This enzyme is known as phosphogluconate dehydrogenase (6PGD), and it is considered to play a role in this malignancy by protecting against decompensated oxidative stress, allowing for more surveillance and improving PCa growth (Gillis et al., 2021).

#### Gluconeogenesis

Gluconeogenesis is a metabolic adaptation pathway that supports other metabolic fuels to be used to generate glucose. Gluconeogenesis is normally regarded to occur in the liver, but new evidence suggests that shortened parts of this pathway occur in cancer cells (Grasmann et al., 2019).

High expression of various gluconeogenic genes, including phosphoenolpyruvate carboxykinase 2 (PCK2), has been identified only in PCa. This enzyme is involved in the first phase of gluconeogenesis, converting oxaloacetate to phosphoenolpyruvate, and its expression is higher in metastatic PCa when compared to *in situ* tumors or normal prostate, implying that glycolysis is reduced in PCa (Grasmann et al., 2019). This gene has also been found to be highly expressed in PCa tumor-initiating cells in metastatic clusters (Grasmann et al., 2019).

Along with this gene, pyruvate carboxylase (PCx) and phosphoenolpyruvate carboxykinase 1 (PCK1) were also reported upregulated in PCa cells (Grasmann et al., 2019).

In general, data suggests that gluconeogenesis points are activated to fulfill the metabolic needs of PCa development and proliferation.

#### Fatty Acid Synthesis

Lipids are essential in the development of PCa, and their significance in cancer is well documented. Although normal cells have a mechanism to inhibit fatty acid synthesis if a determined concentration of fatty acids is present in the blood, PCa cells are insensitive to this mechanism and carry out lipidic biosynthesis due to the increased expression of lipogenesis enzymes regulated by the PI3K/AKT/mTOR, HER2/neu, and AR pathways (Wu et al., 2014). The AMPK pathway appears to play a crucial role in the metabolic shift of PCa, as it promotes glycolysis and inhibits fatty acid production under normal settings, but in the carcinogenic process, fatty acid synthase (FASN) recovers its function by inactivating the AMPK pathway (Kelly et al., 2016; Giunchi et al., 2019).

According to the literature, FASN, one of the most frequently overexpressed metabolic genes in many malignancies, has been linked to increased proliferation, lower apoptosis, and a worse prognosis. This gene’s activity is normally regulated by insulin and nutritional status; however, this regulation is lost throughout the cancer process (Wu et al., 2014). In fact, several authors have described FASN as an oncogene, because its overexpression causes the activation of many other oncogenes (Kelly et al., 2016; Giunchi et al., 2019). Due to its high prevalence in PCa, FASN has been discussed as a potential biomarker and therapeutical target once some data shows a decrease of carcinogenic potential in PCa cell lines when FASN activity is suppressed.

#### β-Oxidation

Even fatty acid production is important in PCa aggressiveness, fatty acid oxidation also contributes to malignancy. Glycolysis is restricted in PCa by a decrease in GLUT1 expression as well as activation of the TCA cycle, as previously documented. As a result, malignant cells rely on β-oxidation to generate the energy required for proliferation and growth (Wu et al., 2014).

#### Cholesterol Metabolism

Cholesterol metabolism appears to be intrinsically connected to PCa for two clinical events: the first is that patients with PCa have a higher incidence of hypercholesterolemia; the second is that administration of statins, a class of drugs that lowers low-density lipoprotein (LDL) cholesterol levels in the blood, improves PCa prognosis (Wu et al., 2014). Activation of the PI3K/AKT/mTOR pathway, together with increased expression of LDL receptors, results in the buildup of cholesteryl ester, a strong inducer of PCa (Kelly et al., 2016).

Another crucial aspect of this pathway is that cholesterol is a source of testosterone synthesis in PCa, which has already been demonstrated to be a key mechanism for increasing aggressiveness (Kelly et al., 2016; Giunchi et al., 2019).

#### Amino Acid Metabolism

Conversion of glutamine into α-ketoglutarate, an intermediate product of the TCA cycle, enables to feed oxidative phosphorylation to produce energy and NAPDH. This metabolic pathway is also important to maintain mitochondrial integrity and nucleotide, protein and lipid synthesis (Kelly et al., 2016; Giunchi et al., 2019).

The glutamine transporters are upregulated by AR activity which causes α-ketoglutarate accumulation. Aside from AR, c-MYC appears also responsible for monitoring the glutamine entrance in TCA and subsequent production of NADPH, which positively influences c-MYC expression, inhibiting apoptosis (Kelly et al., 2016; Giunchi et al., 2019).

**FIGURE 2:**
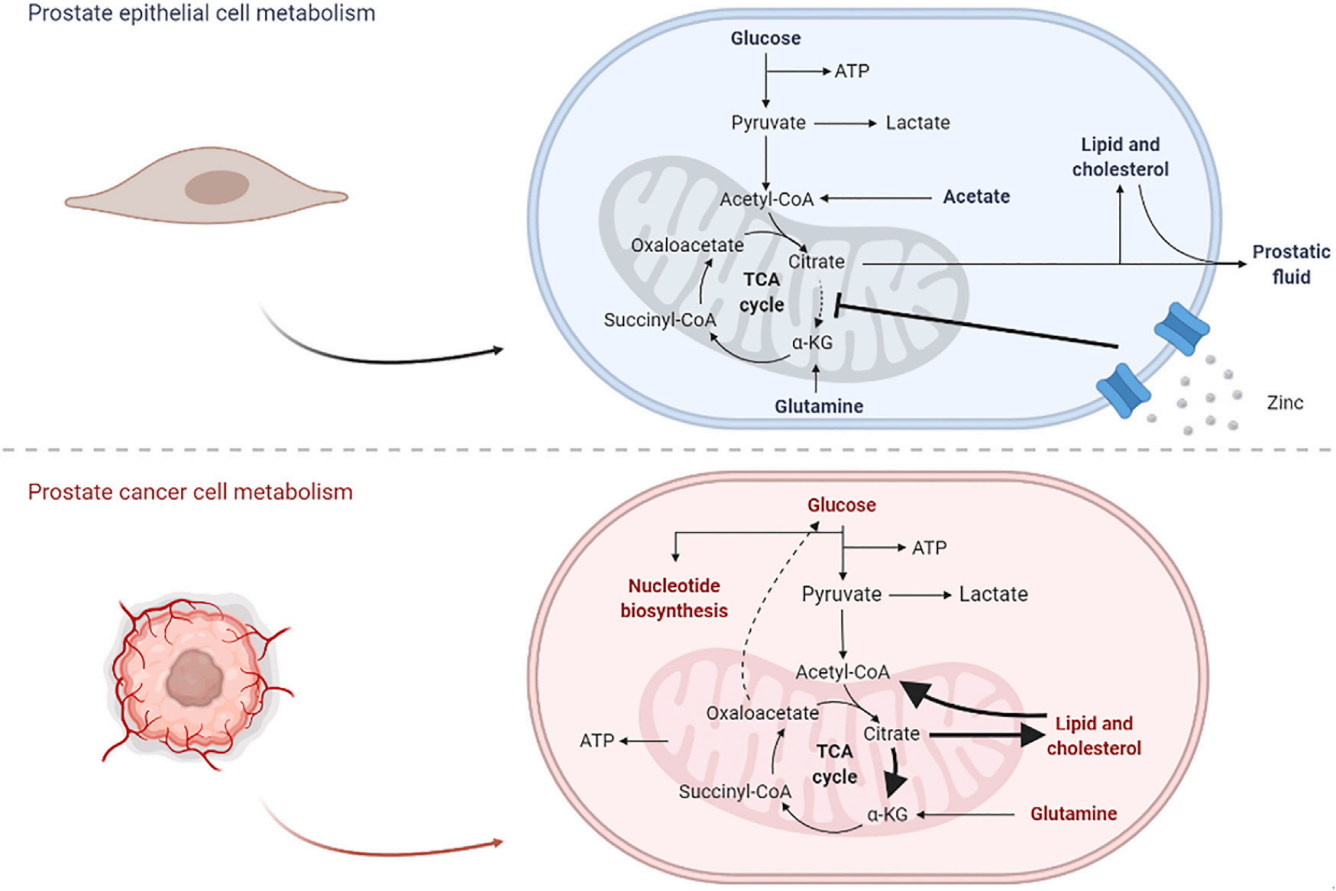
Effects from the carcinogenic process in prostate cell metabolism. In normal epithelial cells, glycolysis plays an important role in the production of citrate, a major constituent of prostatic fluid and lipidic production; once the TCA cycle is disrupted, glutamine is also important for the production of oxaloacetate. In PCa cells, lipid metabolism is overexpressed and citrate is currently proceeding into the TCA cycle, not only for energetic purposes but also for the production of other biomolecules; in parallel, some gluconeogenic steps appear to be activated, in order to maintain normal glucose levels.

This alteration in metabolic mechanisms allows PCa to become more aggressive and proliferative, and the changes between prostate epithelial and PCa cells are depicted in Figure 2.

## Screening and Therapeutic Approaches

To prevent a pandemic progression of PCa, many screening approaches are available, which will be described. There are available numerous classic screening procedures that are commonly used in clinical practice to detect PCa have been described (Sivaraman and Bhat, 2017).

Among traditional methods, one of the most used is the digital rectal examination, and if any abnormalities are found, the assessment of the prostate is performed by a biopsy to obtain a histological diagnosis (with architecture, epithelial-stromal connection, and nuclear dysregulations analysis). This evaluation assigns a value to each histology sample, which is subsequently transformed into a Gleason score based on the tumor’s grade (Bott and Ng, 2015).

Prostate Specific Antigen (PSA) detection is a screening process that uncovers PSA, which is produced by the normal prostate but overexpressed in PCa due to the NKX3.1 mutation, and when levels are greater than 4 ng/ml, a PCa prognosis is established (Sivaraman and Bhat, 2017). Along with PSA concentration, the authors suggest using refined PSA detection methods, such as detecting free and complexed PSA in serum to determine the free: total ratio, which should be less than 25%; another method is to measure PSA density and velocity, which are intrinsically linked to PSA values (Litwin and Tan, 2017; Sivaraman and Bhat, 2017). This set of techniques contributes to the calculation of the prostatic health index, which is a value calculated considering all posterior PSA measurements and is related to the patient’s Gleason score (Sivaraman and Bhat, 2017). Prostate cancer antigen 3 (PCA3) is another overexpressed gene in PCa that can be used as a biomarker alongside PSA values, being detected in urine with a cut-off of 35; a combination of TMPRSS2-ERG/PCA3/serum PSA detection was reported to have a specificity and sensitivity of 90 and 80%, respectively (Litwin and Tan, 2017; Sivaraman and Bhat, 2017). OPPO 4K is a test that uses all of the PSA indicators that have been examined and compares them to digital rectal examination as well as a measurement of human kallikrein 2, which is responsible for promoting the AR pathway (Sivaraman and Bhat, 2017).

In addition to classical methods, radiological approaches have been used to diagnose PCa. One of the most commonly employed approaches is multiparametric magnetic resonance imaging (MRI), which may detect and stage PCa using high-resolution pictures that allow anatomical assessment (Sivaraman and Bhat, 2017). It is also performed in an enhanced version that combines MRI and ultrasound, allowing for a more clinically meaningful and sensitive identification of PCa. Prostate Imaging Reporting And Data Systems (PIRADS) determines the final MRI score, which is a total score of 15 points or less, with punctuation closely tied to the Gleason score (Sivaraman and Bhat, 2017).

Besides the mentioned classical methods, new methodologies have been reported. The first is HistoScanning which evaluates and distinguishes normal prostate tissue from PCa tumors using ultrasound technology. The other method is elastography, which is based on the difference in stiffness between normal and PCa to provide diagnostic information (Sivaraman and Bhat, 2017).

Concerning available treatments to PCa, a set of therapeutical approaches are available to target cancer remission.

When PCa represents a high risk to the host, surgery would be used. This can be a radical prostatectomy, in which all prostate is removed, or a pelvic lymphadenectomy, in which the tumor begins to invade peripheral tissues and must be removed (Chen and Zhao, 2013; Litwin and Tan, 2017). Following surgery, it is suggested that radiotherapy be initiated to prevent and consolidate high-risk PCa treatment, with the most commonly used techniques being external-beam radiotherapy and brachytherapy (Burgess et al., 2021). Proton beam therapy is a type of radiotherapy that, when compared to other radiological procedures, allows for a more specific and accurate radiation dose (Chen and Zhao, 2013). In addition to traditional surgery, cryosurgery has proven to be a suitable and promising therapy option, in which extremely low temperatures are employed to disrupt and destroy PCa cells (Chen and Zhao, 2013).

In aggressive PCa cases, hormonal therapy such as androgen deprivation therapy (ADT) seems to provide a feasible option for this condition. When PCa is in a metastatic stage, ADT is used as a treatment to promote the benefit and quick effects to achieve cancer remission. During ADT, however, cardiac and cognitive impairment were reported (Litwin and Tan, 2017; Wang et al., 2021). Furthermore, chemotherapy is performed in conjunction with ADT to improve life quality and lower PSA blood levels (Chen and Zhao, 2013).

Along with this, the use of metabolic therapeutical approaches has already been under study. FASN inhibition as well as β-oxidation blocking has shown promising efficacy in PCa, once it shifts metabolism into glycolysis activation (Giunchi et al., 2019). The use of statins, as previously described, showed a promising output. The blocking of glutaminase, the first enzyme in glutamine metabolism is also a promising therapeutical target, reducing TCA triggered by glutamine (Kelly et al., 2016).

An individualized therapy appears to be the future of therapeutic procedures. This concept is still in its early stages, but it is based on biochemical analysis of pathology in order to find the most promising molecular targets that will lead to better treatment (Burgess et al., 2021).

Lifestyle adjustments, like those seen in other clinical diseases, may improve PCa prognosis. Adopting a healthy diet and exercise routine, avoiding high fat, cholesterol, and processed foods, and eating more antioxidant rich fruits and vegetables is an effective methods to prevent PCa (Rawla, 2016). Moreover, understanding how other diseases affect PCa progression is a promising therapeutical approach, particularly highlighting metabolic syndrome and diabetes *mellitus*.

## Metabolic Syndrome

Metabolic syndrome (MetS), also known as syndrome X or insulin resistance syndrome, is a group of conditions that raise the risk of acquiring and dying from other diseases such as cancer or diabetes (Reynolds and He, 2005). Obesity, defined by the World Health Organization (WHO) as excessive fat accumulation that poses a risk of death, is a common condition of MetS. It occurs when the energy obtained from metabolizing meal nutrients exceeds the energy required to perform metabolic and physical activity (Reynolds and He, 2005; Marcadenti and de Abreu-Silva, 2015). This excess energy is deposited in lipidic components of adipocytes, increasing the chance of developing a variety of different illnesses such as cardiovascular disease or cancer (Reynolds and He, 2005). The body mass index (BMI) is used to determine the obesity condition, which can be an indicator of MetS. When the value is between 25—29.9 kg/m^2^ it suggests overweight, but when it exceeds 30.0 kg/m^2^ it denotes obesity (Marcadenti and de Abreu-Silva, 2015).

MetS prevalence has increased as a result of the global epidemic of type 2 diabetes mellitus (T2DM) and obesity. Over the last decade, there has been a 27% increase in adulthood and a 47% increase in childhood, and it is expected to reach two-thirds of the adult population in developed countries by 2040 (Avgerinos et al., 2019).

To determine the diagnosis of MetS, three separate classification criteria are used. The first is the presence of glucose > 140 mg/dl (required), with two optional parameters: HDL 0.9 mg/dl in men and 1.0 mg/dl in women; triglycerides > 150 mg/dl; waist circumference > 90 cm in men and 85 cm in women; and blood pressure > 140/90 mmHg (Saklayen, 2018). The National Cholesterol Education Program defines it as possessing three of the following parameters: glucose > 100 mg/dl, HDL 1.0 mg/dl in men and 1.3 mg/dl in women; triglycerides > 150 mg/dl; waist circumference > 102 cm in men and 88 cm in women; blood pressure > 130/85 mmHg (Saklayen, 2018). The International Diabetes Federation considers MetS when waist circumference is obligatorily > 94 cm in men and 80 cm in women, along with two from parameters: glucose > 100 mg/dl, HDL 1.0 mg/dl in men and 1.3 mg/dl in women, triglycerides > 150 mg/dl, and blood pressure > 130/85 mmHg (Saklayen, 2018).

While MetS is typically caused by an unbalanced diet and a lack of physical activity, genetics, and epigenetics appear to play a role. Parental obesity, nutritional dysregulations, or risky behavior by the mother during pregnancy may influence MetS development in the newborn child (Saklayen, 2018). In addition, hypermethylation from IGF2, leptin, and Tumor Necrosis Factor (TNF) appears to exacerbate MetS, altering adipogenesis, glucose homeostasis, and appetite regulation (Saklayen, 2018). Reversion to this condition is achievable with increased physical activity, decreased glucose and cholesterol intake, weight loss, and increased fiber intake (Reynolds and He, 2005).

Normally, this syndrome involves central obesity and an excess of visceral adiposity, which contributes to the development of various disorders. Adipose tissue, in general, causes chronic inflammation, metabolic dysfunction, and immunologic changes that influence DNA repair/apoptosis processes (Torres et al., 2019).

### Adipose Tissue and Metabolic Implications

The MetS mechanism appears to be dependent on adipose tissue control. Several findings indicate pathways in this tissue that are significant in the role of developing this condition. Adipocytes are classified as brown, beige (both with more mitochondrial and thermogenic capacity) and white. Aside from being a reservoir, adipose tissue plays a vital role in the secretion of hormones that regulate metabolism and appetite, such as adiponectin and leptin (Saklayen, 2018). These two specific hormones are antagonists, with leptin decreasing appetite and adiponectin boosting the amount and activity of mitochondria in white adipose tissue, hence regressing MetS (Saklayen, 2018).

Visceral adipose tissue (VAT) is an accumulation of white adipose tissue in the abdominal cavity that poses a significant risk of developing insulin resistance as well as other disorders such as cardiovascular disease and cancer (Zhao, 2013). In energy imbalance, VAT collects triglycerides from the blood, causing hypertrophy and multiplying the tissue, which can be harmful if the growth is uncontrolled owing to a lack of oxygenation and consequent oxidative stress, producing an inflammatory reaction (Marcadenti and de Abreu-Silva, 2015). This inflammatory mechanism is the key to MetS development.

This tissue contains several pro-inflammatory proteins. TNF-α is an acute/chronic inflammatory cytokine generated by M1-macrophages that causes necrosis or apoptosis. It is meaningful not only in MetS development by increasing the release of other pro-inflammatory cytokines such as IL-6 and leptin, but also in T2DM and energy homeostasis (Torres et al., 2019). Other pro-inflammatory proteins seen in obesity and T2DM include IL-1β and IL-6, which not only produce local but also systemic inflammation by activating C-reactive protein in the liver, resulting in inflammatory cascades (Marcadenti and de Abreu-Silva, 2015; Torres et al., 2019). Monocyte chemoattractant protein-1 (MCP-1) is responsible for macrophage infiltration and insulin resistance, and together with Plasminogen activator inhibitor-1 (PAI-1) it promotes atherosclerosis, MetS, and other comorbidities (Zhao, 2013; Torres et al., 2019). Peroxisome proliferator-activated receptor delta (PPAR-δ) appears to be implicated in both adipogenesis and inflammation (Saklayen, 2018). Leptin regulates food intake by directly modulating VAT amount; it also participates in lipogenesis and β-oxidation metabolism, which can enhance oxidative stress and, as a result, inflammation (Marcadenti and de Abreu-Silva, 2015).

Many immune cells and biological mechanisms are active in MetS. This phenomenon involves the presence of cells such as macrophages, T and B cells, eosinophils, dendritic and mast cells (Torres et al., 2019). Macrophages and T cells appear to play a significant role, by their high concentration in this tissue, where CD4+ T lymphocytes upregulate the polarization of M2-macrophage, resulting in an anti-inflammatory response (Marcadenti and de Abreu-Silva, 2015; Torres et al., 2019). B cells cause pro-inflammatory conditions through IgG mediation and IL-10 suppression, whereas dendritic cells secrete IL-6 or 23 and tumor growth factor (TGF-β), which raises the overall state of inflammation (Torres et al., 2019). Mast cells also play an important role in the activation of TNF-α and IL-8, two inflammatory mediators (Torres et al., 2019).

This inflammatory mechanism can cause an increase in blood hypertriglyceridemia, which results in an excess of circulating triglycerides that can be carried to the liver, pancreas, and heart, causing a variety of disorders (Marcadenti and de Abreu-Silva, 2015).

### The Role in Prostate Cancer

MetS plays a critical role in the development of cancer, particularly insulin resistance, obesity, and adipocytokines generated by adipose tissue (Torres et al., 2019). According to Avgerinos et al. (2018) and Gacci et al. (2017), although the mechanisms and metabolic pathways are not well characterized, there are several associations between cancer and the MetS: 1) insulin resistance causes changes in the IGF-1 synthesis and signaling pathway. This factor is involved in cancer cell growth, development, and survival pathways. When changes occur, they increase specific phenomena such as proliferation while decreasing others like apoptosis. Furthermore, they are responsible for an increase in insulin production, which promotes tumor aggressiveness by activating the PI3K/AKT/mTOR pathway, allowing tumor mass to progress (Avgerinos et al., 2019); 2) the overproduction of sexual hormones, due to the biggest proportion of peripheral adipose tissue, causes pathway alterations that increase the likelihood of cancer development. However, data suggests that low testosterone levels (common in obese patients) lead to the formation of less differentiated and more aggressive tumors in some malignancies, such as in high-grade PCa (Gacci et al., 2017; Duarte et al., 2019); 3) accumulation of adipose tissue induces, besides the already mentioned inflammation, oxidative stress and alterations in adipocytokine pathophysiology. This is accomplished by promoting the synthesis of pro-inflammatory hormones like leptin while lowering the production of adiponectin. These two components work together to induce oxidative stress, creating a microenvironment favorable to tumor formation (Gacci et al., 2017; Avgerinos et al., 2019); 4) the gut microbiota is critical for controlling some nutrient metabolism. Evidence suggests that abnormalities in the microbiome can contribute to cancer development and that obese patients can regain their normal weight if their microbiota is normalized (Avgerinos et al., 2019); 5) fluctuations in sleep patterns and abrupt dietary changes can also contribute to the development of cancer in the MetS state (Gacci et al., 2017).

Indeed, all evidence pointed to a substantial link between obesity and cancer, caused not only by well-known factors such as chronic inflammation, but also by insulin resistance, adipokine circuit changes, and microbiome dysregulation (Avgerinos et al., 2019). MetS appear to play a significant influence in practically every cancer formation. Its presence with PCa, for example, appears to amplify high-grade tumors, increasing the chance of death (Gacci et al., 2017).

In PCa, reports suggested that MetS is intrinsically connected to the carcinogenic process, and is thought to be an etiologic predictor of the disease (McGrowder et al., 2012; Gacci et al., 2017), slightly raising the likelihood of developing it (Gacci et al., 2017). One link between both pathologies is the fact that MetS is related to PSA detection, once hemodilution is caused by obesity and decrease of androgens enhances the risk of developing high-grade PCa (McGrowder et al., 2012). Another link is through the IGF-1 pathway, where hyperinsulinemia causes androgen suppression, high levels of estrogen, and a rise in insulin resistance (McGrowder et al., 2012; Gacci et al., 2017). According to several reports, MetS contributes to more aggressive PCa because adipose tissue provides enough fatty acids for PCa to accomplish its metabolism (McGrowder et al., 2012). The aromatase enzyme in VAT, which transforms testosterone irreversibly into estradiol, is one crucial factor in this connection. In more aggressive PCa states, testosterone appears to be converted at a greater rate, causing PCa to progress swiftly and lead to a poor prognosis (McGrowder et al., 2012; Gacci et al., 2017; Duarte et al., 2019). Furthermore, the hypothalamic-pituitary-gonadal axis is disrupted in MetS due to elevated cortisol levels, which restrict androgen synthesis, resulting in a poor prognosis for PCa (McGrowder et al., 2012). This effect generates a cyclical response in which low levels of androgens cause VAT deposition, leading to insulin resistance and severe MetS (McGrowder et al., 2012). MetS’s inflammatory environment is also beneficial in PCa development, with cytokines, growth factors, and other molecules generated by VAT acting as a potential triggering mechanism (Gacci et al., 2017). For this link, it is thought that adiponectin could be employed as a PCa diagnostic biomarker (Bott and Ng, 2015).

## Diabetes Mellitus

Diabetes mellitus (DM) is characterized as a metabolic decompensation that raises blood glucose levels, either by less/non-insulin production by pancreatic β-cells (type 1) or by insulin resistance from cell receptors that require glucose absorption for normal metabolism processes (type 2) (Friedman et al., 2019; Mekala and Bertoni, 2020). In type 2 diabetes (T2DM), chronic hyperglycemia that does not reduce due to insulin insensibility in other tissues results in hyperinsulinemia, which causes failure not just of the β-cell, but also other organ damage and chronic inflammation (Daryabor et al., 2020). Along with the insulin resistance condition, incretins that stimulate insulin secretion appear to be downregulated, resulting in a mechanism known as “the incretin effect” (Mekala and Bertoni, 2020). This kind of DM is deemed high risk since hyperglycemia is a major contributor to other life-threatening comorbidities such as cancer (Javeed and Matveyenko, 2018).

Reports do not discern between T1DM from T2DM due to expensive costs to distinguish them, so the prevalence of T2DM is difficult to measure accurately. Nonetheless, according to the WHO, over 422 million people had diabetes in 2014, with 95% of them having T2DM, and the outlook is for an increase in cases (Javeed and Matveyenko, 2018; Friedman et al., 2019), associated with high rates of death (Mekala and Bertoni, 2020).

Its detection is made recurring to two parameters: the first one is a quick check-up on fasting glucose, considering impaired values above 110 mg/dl; the second one evaluates the effect of glucose of 8–12 weeks in erythrocytes, and is called hemoglobin A1c OR glycosylated (HbA1c), which greater than 6.5 indicates T2DM (Mekala and Bertoni, 2020).

### Etiology and Metabolic Implications

T2DM is a multifactorial and complex disorder that worsens insulin resistance and compromises pancreatic function. Prior to the formation of T2DM, hypertension and growing lipidic accumulation are usually detected and may be exploited to predict its development (Mekala and Bertoni, 2020). Then, insulin resistance develops in some metabolic organs, and if the skeletal muscle is compromised, blood glucose accumulates as this is the primary organ that regulates postprandial glucose (Javeed and Matveyenko, 2018).

The mechanism of insulin resistance includes failure to uptake glucose into the cell by GLUT4 followed by decreased glucose metabolism, incapacity of glycogen storing and dysfunctional mitochondria (Javeed and Matveyenko, 2018). The liver is also affected by this impairment, and as insulin is not being recognized, it starts gluconeogenesis to produce glucose. Adipose tissue is affected as well, leading to constant lipolysis and chronic inflammation with high levels of fatty acids and adipokines in blood, which are responsible for β-pancreatic cell disruption (Javeed and Matveyenko, 2018).

Some risk factors have been linked to this metabolic disorder. As with MetS, old age, sedentary lifestyle, overweight, drinking alcohol, smoking, and having a poor diet all contribute to the development of T2DM (Mekala and Bertoni, 2020). In addition, some studies have suggested that genetic and epigenetic factors play a role in T2DM. A family history of T2DM indicates a genetic vulnerability, with numerous loci identified (Mekala and Bertoni, 2020). Even though these alterations have received slight investigation, T2DM high rising prevalence seems to be caused by them (Javeed and Matveyenko, 2018).

Hyperglycemia is unquestionably one of the most visible clinical signs of T2DM development. On a macro scale, it can cause nephropathy, neuropathy, and retinopathy; on a cellular level, it causes mitochondrial dysregulation, which produces more reactive oxygen species (ROS) than typical, resulting in mitochondrial damage accumulation and, ultimately, cell aging (Daryabor et al., 2020). This mechanism is observed in β-pancreatic cells, resulting in cell death and, as a result, insulin synthesis is disrupted (Daryabor et al., 2020). Furthermore, the connection between macromolecules and ROS results in the creation of advanced glycation end products, which appear to be significant in activating the immune system through the release of cytokines and other pro-inflammatory proteins (Daryabor et al., 2020).

The vast majority of T2DM patients also have dyslipidemia, an inflammatory process in which an excess of metabolic substrates causes metabolic organs, such as the liver, to overproduce triglycerides and low-density lipoprotein (LDL) while decreasing the production of high-density lipoprotein (HDL), thus linking this pathology to cardiovascular disease (Parhofer, 2015). When glucose levels are regulated, dyslipidemia appears to improve (Parhofer, 2015).

This dysfunction is seen not just in T2DM, but also in MetS, and it is thought that both disorders have a similar pathogenic genesis. Insulin resistance, dysregulated glucose metabolism, increased adipose tissue and disrupted lipidic utilization in skeletal muscle are the key links between these two metabolic abnormalities (Parhofer, 2015; Antunes et al., 2018). Furthermore, deficits in glucose-6-phosphatase, the final enzyme in gluconeogenesis, suggest a difficulty with transport or phosphorylate glucose (Blaak, 2003). As a result, MetS patients are more likely to develop T2DM (Daryabor et al., 2020).

### The Paradox: PCa vs. T2DM

Some data suggest that patients with T2DM and metabolic syndrome are more likely to acquire some malignancies, such as those of the gastrointestinal system, breast, bladder, endometrial, and colon, allowing them to grow and become more aggressive (Antunes et al., 2018; Gillies et al., 2019). Some T2DM hazards have been linked to cancer morbidity and mortality, with the most prevalent being a lack of physical exercise, smoking behaviors, age, and recurrent/excessive alcohol usage (Gillies et al., 2019). T2DM also creates a microenvironment with elevated glycation products and hyperlipidemia, which can promote tumor growth. Chronic local inflammation is also another feature that links T2DM to cancer, which provides an ideal environment for cancer formation. Nonetheless, this mechanism is still poorly understood (Griffiths et al., 2018; Gillies et al., 2019).

Regardless, the fundamental aspect that links cancer to T2DM is the reliance on glucose fermentation, which produces high levels of lactate and leads to local acidosis, either in T2DM or in cancer. The Warburg effect describes the mechanisms of metabolic transition from aerobic metabolism to glucose fermentation (Luis et al., 2019). This acidosis promotes inflammation status, which improves the tumoral microenvironment, allowing invasion, metastasis, tissue remodeling, and immune system suppression to occur. Additionally, it causes genetic instability and the selection of more aggressive cancers (Gillies et al., 2019).

Nevertheless, these effects of T2DM are not transversal for every type of cancer. In fact, T2DM has been described, mainly by epidemiological research, to be a protective factor against PCa (Crawley et al., 2018); in another hand, the fact that T2DM is commonly linked with MetS and/or obesity (that was previously described to enhance the aggressiveness of PCa) suggests that this plethora of metabolic disturbs should increase the aggressiveness of PCa (Mekala and Bertoni, 2020). The outcome of these two mechanisms creates a paradox concerning prostate cancer, T2DM and MetS, where T2DM seems to inversely correlated with the aggressiveness of PCa, and seems to overlay the effect of MetS (Crawley et al., 2018).

Although the paradox is present in the literature, the link between PCa and T2DM is highly debatable. Several reports, albeit not unanimous, support the hypothesis that these disorders are inversely connected (Antunes et al., 2018). T2DM is independent of PCa development, according to Gacci et al. (2018) and Antunes et al. (2018). Indeed, studies have shown that men without diabetes have a higher risk of developing low-grade tumors than diabetic patients, and T2DM males have a lower risk of developing PCa than non-T2DM patients, with the addition that high-grade tumors are more common in individuals who do not have T2DM (although it is not consistent with all literature) (Rastmanesh et al., 2014; Miller et al., 2018).

From a molecular standpoint, the literature is limited, however, some data indicate hyperglycemia as the cause of this paradoxical impact, with probable mechanisms signaling IGF-1, inflammation, SRF5A2, and AR pathways (Bansal et al., 2013; Antunes et al., 2018). AR activation caused by hyperglycemia activates the AMPK pathway, resulting in glycolysis and lipogenesis activation; it also plays a role in HER/neu pathway activation, which increases aggressiveness and leads to a poor prognosis in PCa; however, an increase in GLUT1 expression was also observed in PCa, which may reduce hyperglycemia state (Antunes et al., 2018). Moreover, the hypothalamic-pituitary-testicular axis appears to be negatively altered in T2DM, influencing PCa development (Bansal et al., 2013). Additionally, T2DM causes a drop in insulin during disease staging, which is crucial considering insulin is an important growth factor in PCa progression; also, it appears to alter the IGF-1 pathway, which is an important mechanism in PCa to boost cell proliferation rate (Bansal et al., 2013; Rastmanesh et al., 2014).

Genetics appears to have a significant impact on PCa. In fact, patients with a high susceptibility to T2DM development had a decreased risk of developing PCa, and certain diabetes polymorphisms in JAZF1 and HNF1B genes appear to be adversely connected with the formation of PCa (Bansal et al., 2013; Rastmanesh et al., 2014).

Less PSA levels may potentially play a role in the “protector” mechanism, not only because it is engaged in lower androgen concentrations, but also because it may be undetectable due to hemodilution produced by recurrent obesity in T2DM (Antunes et al., 2018; Miller et al., 2018). Lower androgens induced by T2DM have also been shown to be a preventive strategy against PCa due to decreased AR pathway activation (Bansal et al., 2013; Rastmanesh et al., 2014).

Although additional research is needed, leptin appears to influence the occurrence of PCa in T2DM patients. This hormone increases the likelihood of developing PCa, and its levels in T2DM are significantly lower than in MetS (Rastmanesh et al., 2014).

The microvascular environment has also been suggested as a possible explanation for the paradox. T2DM patients have complications in the angiogenesis process, and as a result, PCa may be less aggressive in ischemia, because its metabolism is reliant on lipidic reactions that consume oxygen in mitochondria (Rastmanesh et al., 2014). The systemic inflammatory environment observed in T2DM also benefits the protective effect in PCa, due to TNF-α cascade and nuclear factor κB (NF-κB) (Barbosa-Desongles et al., 2013).

Another feature is the fact of some T2DM medications seems to present benefit activity against PCa. Despite contradictions in the literature, reports link preventive effects of metformin, for example, against PCa; nevertheless, the administered dose appears to impact the effect in PCa (Rastmanesh et al., 2014; Antunes et al., 2018; Au Yeung and Schooling, 2019; Yuan et al., 2020). Some other T2DM medications are under study, and their effects are still unknown.

C-X-C Motif Chemokine Ligand 14 (CXCL14), a dendritic recruiter that is activated in specific circumstances such as obesity and insulin resistance, is another possibility that can explain this behavior. Once systemic insulin resistance is observed in advanced stages of T2DM, the CXCL14 gene may become more active in PCa, resulting in its regression through immune response (Rastmanesh et al., 2014).

## Conclusion

PCa is the world’s second most common cancer in men. The need for novel therapeutics is urgent and preventing risk factors may be a useful approach. Unlike in other malignancies, T2DM appears to have an antitumor effect on PCa prognosis, even though MetS, a common comorbidity of diabetes, appears to have the opposite effect. Thereby, these mechanisms need to be further explored to shed light on new preventive and therapeutical approaches to ameliorate the outcome of PCa patients.

## Future Perspectives

*In vitro* studies based on prostate cell lines (epithelial and cancer cell lines) could be employed as a model to study cancer metabolic state and development. This model could be applied to study the influence of physiological environments of T2DM and MetS and their effect in PCa. Furthermore, research into the circulating factors of each of metabolic disorders, as well as an understanding of their effect on tumor cells, may be significantly relevant and lead to novel therapeutic interventions.
